# Two Novel 30K Proteins Overexpressed in Baculovirus System and Their Antiapoptotic Effect in Insect and Mammalian Cells

**DOI:** 10.1155/2013/323592

**Published:** 2013-06-03

**Authors:** Wei Yu, Qing Li, Yuhua Yao, Yanping Quan, Yaozhou Zhang

**Affiliations:** ^1^Institute of Biochemistry, Zhejiang Sci-Tech University, Hangzhou 310018, China; ^2^Zhejiang Provincial Key Laboratroy of Silkworm Bioreactor and Biomedicine, Hangzhou 310018, China

## Abstract

The 30K family of proteins is important in energy metabolism and may play a role in inhibiting cellular apoptosis in silkworms (*Bombyx mori*). Several 30K-family proteins have been identified. In this study, two new silkworm genes, referred to as *Slp* (NM 001126256) and *Lsp-t *(NM 001043443), were analyzed by a bioinformatics approach according to the sequences of 30K proteins previously reported in the silkworm. Both Slp and Lsp-t shared more than 41% amino acid sequence homology with the reported 30K proteins and displayed a conserved domain consistent with that of lipoprotein-11. Additionally, the cDNA sequences of both *Slp *and *Lsp-t* were obtained from the fat bodies of silkworm larvae by reverse transcription polymerase chain reaction. Both genes were expressed in BmN cells using the Bac-to-Bac system. Purified Slp and Lsp-t were added to cultured BmN and human umbilical vein endothelial cells (HUVEC) that were treated with H_2_O_2_. Both Slp and Lsp-t significantly enhanced the viability and suppressed DNA fragmentation in H_2_O_2_ treated BmN and HUVEC cells. This study suggested that Slp and Lsp-t exhibit similar biological activities as their known 30K-protein counterparts and mediate an inhibitory effect against H_2_O_2_-induced apoptosis.

## 1. Introduction

The 30K-family proteins are lipoproteins synthesized by the fat body in the silkworm and display a molecular weight of approximately 30kD [1–3]. They are highly expressed in silkworm larvae during the early stage of the fifth instar and during the pupation stage of development, where they are predominantly located in the hemolymph as a major source of energy for growth, development, and pupation. In addition, the 30K-family proteins found in the silkworm are highly conserved, share more than 41% amino acid sequence homology, and display similar tertiary structures with the common conserved domain of lipoprotein-11 [4].

Moreover, the 30K family of proteins was found to have an antifungal activity [5]. Ujita et al. found that the purified recombinant glutathione-fused 30K protein Lp6G1 inhibited the morbidity of silkworm pupae and prolonged the life of the fungus-infected silkworm chrysalis [5]. Thus far, several 30K proteins were also reported to have an antiapoptotic activity [6–11]. Park et al. demonstrated that the purified 30Kc6 protein that was expressed in *Escherichia coli*, enhanced cell viability, and inhibited apoptosis of both virus-infected Sf9 cells and chemical-treated HeLa cells [6]. Rhee et al. discovered that the purified *Escherichia coli* expressed 30Kc19 protein enhanced cell viability and prevented cell apoptosis in actinomycin-induced Sf9 cells [8]. Wang et al. showed that 30Kc19 expressing Chinese hamster ovary cells increased both the quantity and biological activity of human erythropoietin [9]. Previous studies by us have shown that the purified 30K proteins expressed in the insect system encoding 30Kc6, 30Kc12p, and 30Kc19G1 inhibited apoptosis in H_2_O_2_-induced human umbilical vein endothelial cells (HUVEC) [7]. 

In the current study, both *Slp* and *Lsp-t* were initially screened from a gene library of the silkworm by comparative homology, where it was found that both *Slp* and *Lsp-t* shared a high degree of amino acid sequence homology and a predicted tertiary structure consistent with the reported silkworm family of 30K proteins. Furthermore, *Slp* and *Lsp-t* were cloned, expressed, and purified. In novel experiments, we studied the effects of Slp and Lsp-t on H_2_O_2_-induced apoptosis silkworm BmN cells and HUVEC cells by analyzing both the cell viability and mechanisms of cell death. 

## 2. Materials and Methods

### 2.1. Materials

The *E. coli* strain TG1, the DH10Bac strain, the pFastBac HTb vector, and cultured silkworm BmN cells were conserved in our laboratory. HUVEC cells were purchased from ATCC (Manassas, VA, USA). A Nikon TE2000-E microscope was purchased from Nikon Corporation (Japan). A Tanon-1600 gel image system was purchased from Shanghai Tianneng Science and Technology Limited Company (Shanghai, China). Trizol and RT-PCR kits were purchased from Promega (USA). Mouse 6 × His-tag monoclonal antibody and the Cell Death Detection ELISA kit were purchased from Roche Life Science, Inc., (USA). Horseradish peroxidase (HRP)-conjuagated goat-antimouse-IgG was purchased from Beijing Dingguo Biotechnology Company (Beijing, China). Serum-free insect cell culture medium SF-900II SFM, mammalian high glucose culture medium DMEM, fetal bovine serum (FBS), and Hank's balanced salts solution (HBSS) buffer were all obtained from Gibco/Invitrogen (USA). The liposomal transfection reagent, Cellfection II Reagent, and the Ni-NTA Purification System were also obtained from Invitrogen (USA). 

### 2.2. Sequence Analysis and Gene Cloning of *Slp* and *Lsp-t*

Silkworm Slp and Lsp-t were screened, and their cDNA sequences were obtained by the online available BLAST tool (http://blast.ncbi.nlm.nih.gov/). The cDNA open reading frames (ORF) of the targeted genes were obtained by the DNAStar software program. DNA sequences analysis of *Slp* and *Lsp-t* and homology comparisons with three reported 30K-family proteins, including 30Kc6 [6, 12, 13], 30Kc12p [7], and 30Kc19G1 [8, 9] were carried out using the clustal method of MegAlign in DNAStar. Homology comparisons of the amino acid sequences present in Slp and Lsp-t were conducted by the single version ClustX1.8 and BioEdit software programs according to the protein sequences and functions of the three reported 30K-family proteins. SWISS-MODEL (http://swissmodel.expasy.org/) and RasMol Version2.7 were employed to predict the tertiary structures. PCR primers were designed according to the screened sequences of both *Slp* and *Lsp-t* as shown below:

P1: 5′-CACGGATCCATGAAGTTTTTGGTGTTTTTC-3′;
P1′: 5′-CCCAAGCTTTTAGTTGAGGATTGAGATAGAC-3′
P2: 5′-ATCGGATCCATGAAGACCTTGGCGG-3′
P2′: 5′-CGCAAGCTTTTAGTTGAGGATCGAGATAG-3′. Note that the *Bam*H I and *Hind* III restriction enzyme digest sites were introduced and indicated by the underlined text.

Total RNA was extracted from the fat body of a 5th instar silkworm larvae by grinding the collected tissues in liquid nitrogen. cDNA was obtained using an RT-PCR kit and by using the P1/P1′ and P2/P2′ primers, respectively. Both *Slp* and *Lsp-t* were amplified, purified and then cloned into T-easy vectors, respectively. The recombinant plasmids were identified by restriction enzyme analysis using *Bam*H I and *Hind* III. 

### 2.3. Construction of Recombinant Bacmid-*Slp* and Bacmid-*Lsp-t*

The constructed T-easy vectors including sequences of both *Slp* and *Lsp-t*, and the transfer vector pFastBacHTb, were digested with the same restriction enzymes described above and ligated. *E. coli* DH10Bac competent cells were transformed with the constructed recombinant transfer vectors and subsequently cultured on LB media containing IPTG, X-gal, gentamycin, kanamycin, and tetracycline. Positive clones were identified by PCR using forward and reverse M13 primers: forward primer is 5′-CCCAGTCACGACGTTGTAAAACG-3′.

Reverse primer is 5′-AGCGATAACAATTTCACACAGG-3′. The screened recombinant DNA for Bacmid-*Slp* and Bacmid-*Lsp-t* was transfected into BmN cells by a liposome-mediated method and the virus titer determined by the TCID_50_ (50% tissue culture infective dose) cytopathic effect endpoint dilution assay, which quantifies the amount of virus required to kill 50% of infected cultured host cells. 

### 2.4. Expression and Purification of the Recombinant *Slp* and *Lsp-t* Proteins

BmN cells were infected with the harvested second generation viruses at a multiplicity of infection (MOI) of 10. The infected cells were harvested 72 h following viral infection and washed once with PBS. Cells were resuspended in PBS and lysed by an ice-bath ultrasonic sound method. The lysates were centrifuged for 15 min at 12000 g under 4°C using a Beckman centrifuge (A1330011), and the supernatants were harvested and incubated with a Ni-NTA agarose affinity filler on a platform orbital shaker overnight. The collected samples were washed in 20 mM imidazole and 250 mM imidazole three times, respectively. The purification of proteins was conducted according to the manufacturer's instructions (Invitrogen) of the Ni-NTA kit. A mouse 6 × His-tag monoclonal antibody was used to identify the target proteins by Western immunoblotting.

### 2.5. Cell Viability and Analysis of Apoptosis in H_**2**_O_**2**_-Treated BmN Cells

#### 2.5.1. H_**2**_O_**2**_ Induced Apoptosis in BmN Cells

BmN cells were used in the logarithmic growth phase and plated in 96-well microtiter plates at a density of 1 × 10^3^ cells/well and cultured overnight. Appropriate dilutions of H_2_O_2_ were made in HBSS buffer at 0.1 mM, 0.5 mM, and 1 mM, respectively. The cell culture media were discarded from the 96-well plates, which were washed once with HBSS prior to adding the appropriate dilutions of H_2_O_2_ to the wells. H_2_O_2_-treated cells were cultured for 10 min, 30 min, or 60 min in an incubator set at a temperature of 27°C. H_2_O_2_ was discarded, and the cells were washed twice with HBSS. Finally, cells were added to regular culture media, supplemented with FBS with a 10% final concentration for 24 h at 27°C.

#### 2.5.2. Treatment of BmN Cell with Slp and Lsp-t

BmN cells in the logarithmic phase of growth were plated in 96-well microtitre plates at a density of 1 × 10^3^ cells/well and cultured overnight at 27°C. Cultured cells were pretreated with purified Slp and Lsp-t at a final concentration of 1.5 *μ*g/mL for 24 h. BmN cells were then treated with 0.5 mM H_2_O_2_ for 10 min to induce apoptosis, following which, cells were cultured in complete media containing Slp and Lsp-t for an additional 24 h. Purified 30Kc6 protein was used as a positive control. Normal cultured BmN cells and H_2_O_2_-treated BmN cells served as control groups. Each group was repeated three times. 

#### 2.5.3. Evaluation of Cell Vitality

The treated BmN cells were pulsed with 10 *μ*L of MTT reagent (5 mg/mL) per well of a 96-well microtitre plate and cultured for 4 h in the incubator and in the dark at 27°C. Next, the culture media was discarded, and 150 *μ*L of DMSO was added to each well to a final concentration of 0.1%. After shaking for 10 min, the absorbance of each well was measured at a wavelength of 490 nm to calculate the relative cell viability ratio.

#### 2.5.4. Evaluation of Cellular Apoptosis

For quantitative determination, apoptosis of BmN cells was measured as DNA fragmentation. DNA fragmentation was evaluated by histone-associated DNA fragments using a photometric enzyme immunoassay (Cell Death Detection ELISA, Roche Life Sciences, Inc.), according to the manufacturer's instructions. The treated BmN cells were harvested, and the cell suspension was pelleted by centrifugation. Floating and attached cells were lysed. After centrifugation, 10 *μ*L volumes of the supernatants were added to streptomycin-coated culture plates. Each culture plate was then incubated with 80 *μ*L of specific antibody for 2 h. After washing three times in PBS, samples were measured by an ELISA format procedure and spectrophotometry to read the absorbance values of each well. 

### 2.6. Cell Viability and Apoptosis Analysis in H_**2**_O_**2**_-Induced HUVEC

#### 2.6.1. H_**2**_O_**2**_ Induced-Apoptosis in HUVEC

HUVECs were propagated to the logarithmic growth phase, seeded into 96-well microtiter plates at a density of 1 × 10^3^ cells/well, and cultured overnight at 37°C with 5% CO_2_. The cells were washed once in HBSS and stimulated with different concentration of H_2_O_2_ (0.25 mM, 0.5 mM, and 1.0 mM) for 10 min, 30 min, or 60 min in an incubator set at a temperature of 37°C. Culture media containing H_2_O_2_ were discarded, and cells were washed twice in HBSS buffer. Finally, cells were cultured in normal culture media with 10% FBS in the incubator with 5% CO_2_ for 24 h at 37°C. 

#### 2.6.2. Treatment of HUVEC with Slp and Lsp-t

HUVEC cells were pretreated with Slp and Lsp-t as described previously, following which, cells were treated with 0.5 mM H_2_O_2_ for 30 min. Next, the cells were incubated with either Slp or Lsp-t at a final concentration of 4 *μ*g/mL and cultured for 24 h. Control groups were set up exactly as described for the cultured BmN cells. Cell viability in the treated HUVEC cells was conducted exactly as described previously and by the MTT reagent assay. DNA fragmentation analysis in HUVEC cells was conducted as described previously by an ELISA assay.

### 2.7. Statistical Analysis

Data described in this paper are shown as mean ± SD. Statistical significance between means was determined and analyzed by one-way analysis of variance (ANOVA) and Student's *t*-test using SPSS version 13.0 software. A *P* value <0.05 was considered statistically significant, and a *P* value <0.01 was considered highly significant.

## 3. Results

### 3.1. Comparison of the Homology between *Slp* and *Lsp-t*

**Figure 1:**
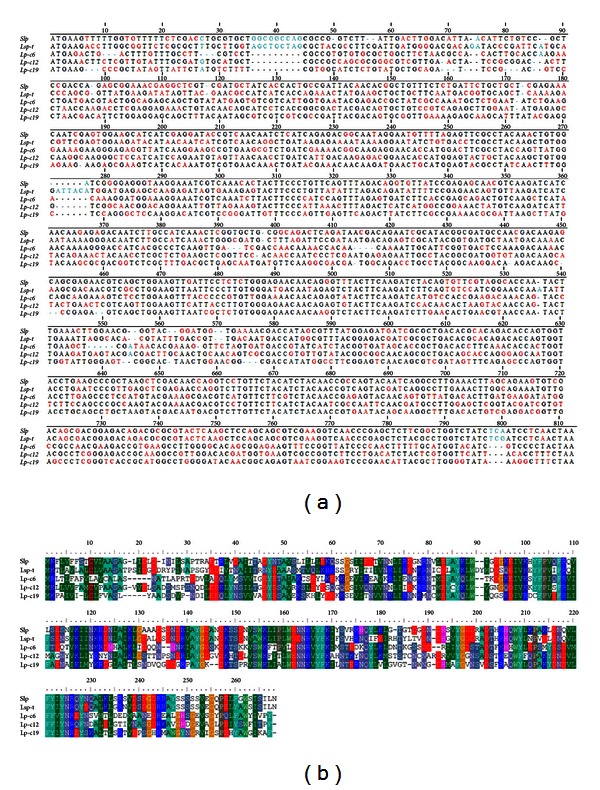
Comparative homology analysis of DNA sequences (a) and amino acid residues (b) of silkworm 30K proteins. Slp: silkworm Slp protein; Lsp-t: silkworm Lsp-t protein; Lp-c6: silkworm lipoprotein 30Kc6; Lp-c12: silkworm lipoprotein 30Kc12p; Lp-c19: silkworm lipoprotein 30Kc19G. Conserved domains of lipoprotein-11: Slp (amino acids 6–261), Lsp-t (amino acids 6–266). Conserved amino acid residues are marked as same color.

**Figure 2:**
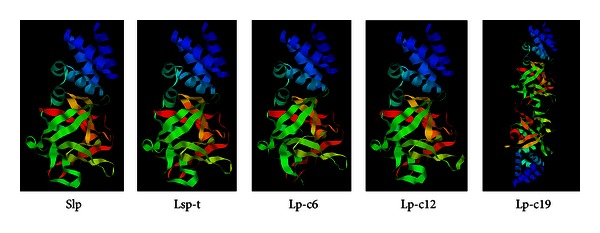
Predicted tertiary structures of the 30K-family proteins in the silkworm. Slp: silkworm Slp protein; Lsp-t: silkworm Lsp-t protein; Lp-c6: silkworm lipoprotein 30Kc6; Lp-c12: silkworm lipoprotein 30Kc12p; Lp-c19: silkworm lipoprotein 30Kc19G. The blue color describes the alpha helix, and the green color indicates the beta-pleated sheets.

The 30K family of proteins is structurally and functionally conserved. There are only several 30K-family proteins reported to be expressed in the silkworm. To identify new members of the 30K family of proteins, a gene library derived from the silkworm was screened with the clustalx 1.8 software program by comparative homology analysis against the reported 30K family of proteins (Figure 1). This analysis revealed that both Slp and Lsp-t shared more than 41% homology with the already characterized 30K family of proteins (Figure 1(b)). Furthermore, there were conserved domains of lipoprotein-11 in both Slp (amino acids 6–261) and Lsp-t (amino acids 6–266) and the other reported 30K family of proteins. Tertiary structures of both Slp and Lsp-t were further predicted by applying SWISS-MODEL software (Figure 2), which showed that the predicted tertiary structures of Slp and Lsp-t were similar to that of the reported 30K family of proteins. These data indicate that both Slp and Lsp-t are novel candidates of the 30K family of proteins in the silkworm. 

### 3.2. PCR Identification of the Recombinant Bacmid-*Slp* and Bacmid-*Lsp-t* Constructs

**Figure 3:**
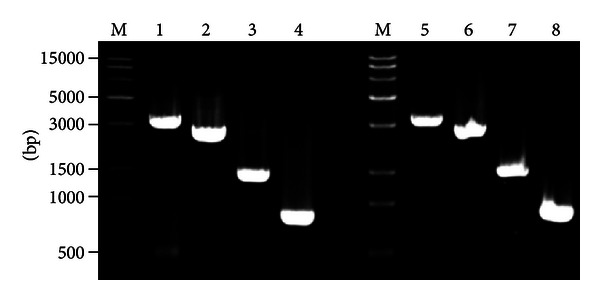
Cross-amplification PCR identification of recombinant Bacmid-*Slp* and Bacmid-*Lsp-t. *M represents the DNA molecular weight marker. Lanes 1–4 represent cross-amplification PCR products of the recombinant Bacmid-*Slp* with M13F and M13R, M13F and P1′, M13R and P1, and P1 and P1′ as primers, respectively (see Section 2). Lanes 5–8 represent cross-amplification PCR products of the recombinant Bacmid-*Lsp*-*t* with M13F and M13R, M13F and P2′, M13R with P2, and P2 and P2′ as primers, respectively.

In order to identify the recombinant Bacmid, we extracted DNA of the recombinant Bacmid and subjected it to PCR analysis using M13 primers and the targeted *Slp* and *Lsp-t* gene primers, respectively. The cross PCR results demonstrated that there were four PCR products with sizes of 3220 bp, 2620 bp, 1480 bp, and 789 bp in the analysis of Bacmid-*Slp* (Figure 3). In addtion, there were four PCR products with sizes of 3230 bp, 2630 bp, 1490 bp, and 804 bp in the analysis of Bacmid-*Lsp-t* (Figure 3). These data were consistent with the predicted results and thus indicated that both recombinant Bacmid-*Slp* and Bacmid-*Lsp-t* were correct and could be used in further functional studies. 

### 3.3. Identification of the Expression and Purification of Recombinant Slp and Lsp-t

**Figure 4:**
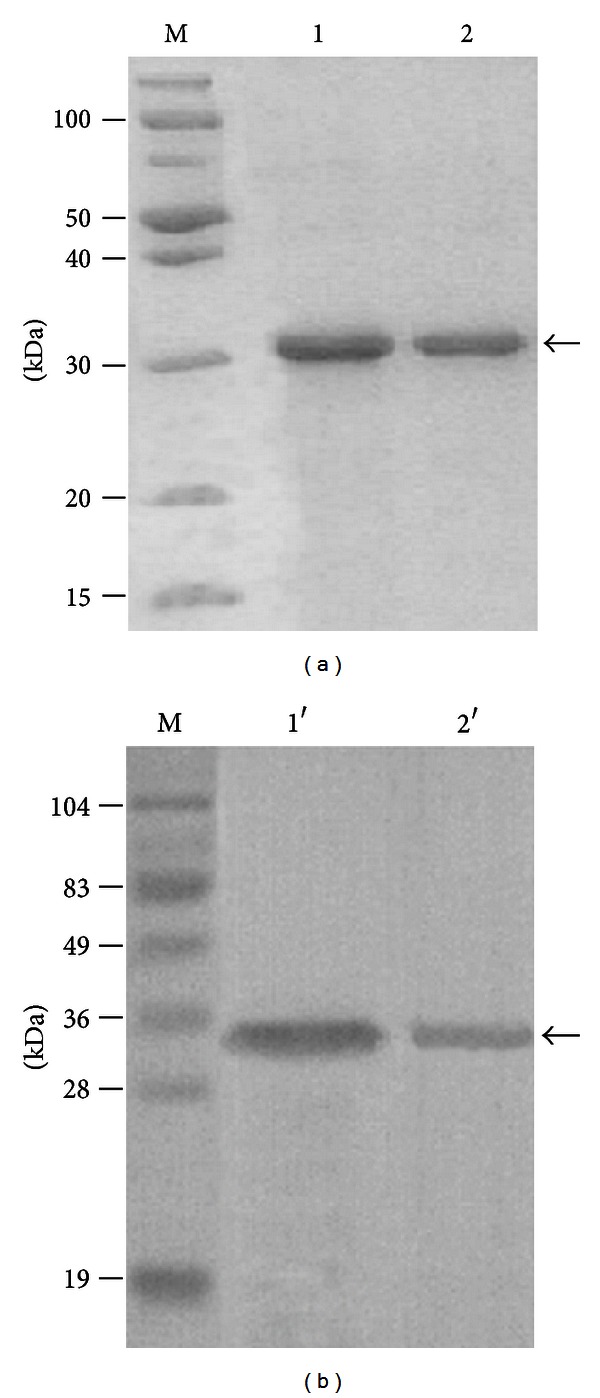
Identification of the recombinant Slp and Lsp-t proteins by SDS-PAGE (a) and Western blot (b). M represents the protein molecular weight marker. Lane 1 represents the purified recombinant Slp, and lane 2 represents the purified recombinant Lsp-t proteins by SDS-PAGE analysis. Lanes 1′ and 2′ represent the purified recombinant Slp and Lsp-t proteins as determined by Western immunoblotting analysis. Arrows indicate the target bands.

In order to study the function of Slp and Lsp-t, recombinant Bacmid-*Slp* and Bacmid-*Lsp*-*t* were transformed into BmN cells. Next, the BmN cells that expressed the Bacmid-*Slp* and Bacmid-*Lsp*-*t* constructs were lysed, and the supernatants were collected and purified with the Ni-NTA procedure according to the kit directions. The eluted proteins were harvested and subjected to SDS-PAGE and Western immunoblotting analysis (Figure 4). This analysis showed that both of the recombinant Slp and Lsp-t (Figure 4(a)) proteins and the hybridized protein bands (Figure 4(b)) appeared in the predicted positions. 

### 3.4. Effects of Recombinant Slp and Lsp-t on Cell Viability and Apoptosis in H_**2**_O_**2**_-Induced BmN Cells

**Figure 5:**
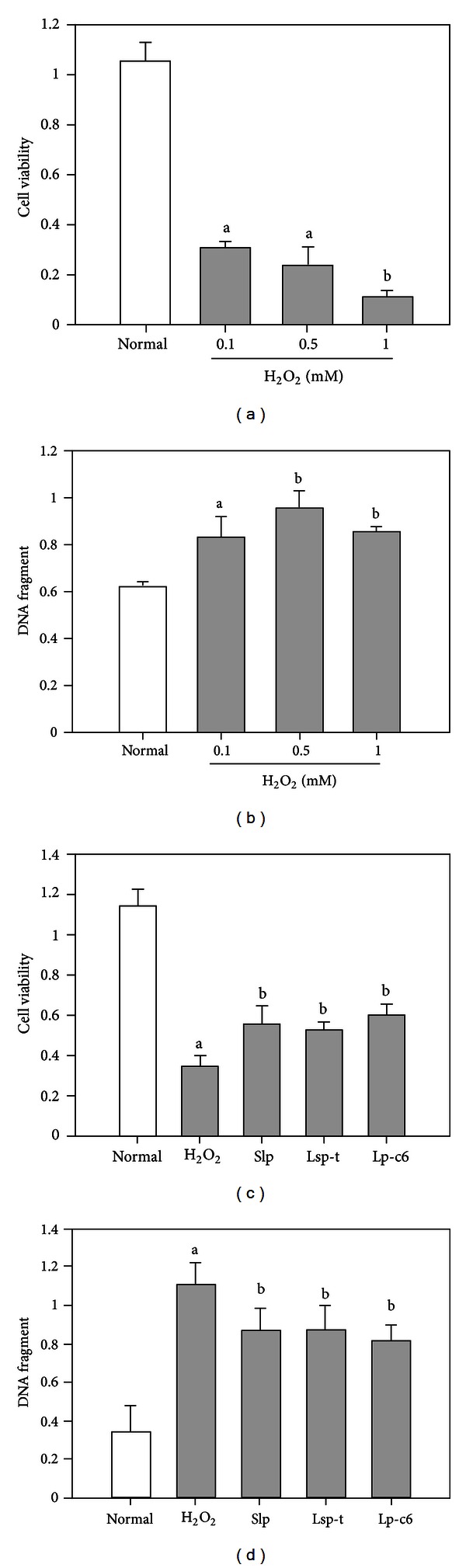
Effects of Slp and Lsp-t on H_2_O_2_-treated BmN cells. (a) Analysis of BmN cellular viability on exposure to different concentration of H_2_O_2_ as determined by MTT assay. (b) DNA fragmentation analysis on exposure of BmN cells to different concentration of H_2_O_2_ as determined by ELISA. (c) Effects of Slp and Lsp-t on cell viability on exposure to different concentrations of H_2_O_2_ as determined by MTT assay. (d) Effects of Slp and Lsp-t on DNA fragmentation in H_2_O_2_-treated BmN cells by ELISA. Purified silkworm lipoprotein 30Kc6 (Lp-c6) was used as a positive control. Different letters refer to the levels of statistical significance where there was a significant difference at the *P* < 0.05 level. Where the letters match, this refers to no statistical difference between the sets of data.

It has been previously shown that the 30K-family proteins in the silkworm display antiapoptotic activity, especially 30Kc6 [3, 8, 14, 15]. However, whether the newly predicted Slp and Lsp-t proteins inhibit cellular apoptosis remains undetermined. Thus, to study the effects of Slp and Lsp-t on cellular apoptosis, increasing concentrations of H_2_O_2_ were applied to the cells to examine the effects of such exposure on BmN cellular apoptosis. Based on time-dependent experiments (data not shown), the viability and apoptosis of BmN cells were determined 24 h after a brief 10 min treatment in the presence of H_2_O_2_. Dose-dependent effects of H_2_O_2_ on BmN cellular activity (Figure 5(a)) and apoptosis were also determined (Figure 5(b)). However, we did not see any further significant increase in cellular apoptosis of BmN cells following treatment with H_2_O_2_ a dose greater than 0.5 mM. Based on these data, the following experiments were pursued using 0.5 mM H_2_O_2_. In these experiments, cultured BmN cells were preincubated with purified recombinant Slp or Lsp-t for 24 h and then treated with 0.5 mM H_2_O_2_ for 10 min. Cells were then additionally treated with Slp or Lsp-t for 24 h. Purified 30Kc6 protein was used as a positive control. We found a statistically significant difference in respect to cell viability (Figure 5(c)) and DNA fragmentation (Figure 5(d)) between H_2_O_2_-treated cells and those cells treated with Slp, Lsp-t, and 30Kc6. Taken together, these data indicated that both recombinant Slp and Lsp-t have similar biological activities to 30kc6 protein. They could enhance cell viability, decrease DNA fragmentation, and inhibit cell apoptosis following treatment of BmN cells to H_2_O_2_. 

### 3.5. Effects of Recombinant Slp and Lsp-t on Cellular Viability and Apoptosis in H_**2**_O_**2**_-Induced HUVEC

**Figure 6:**
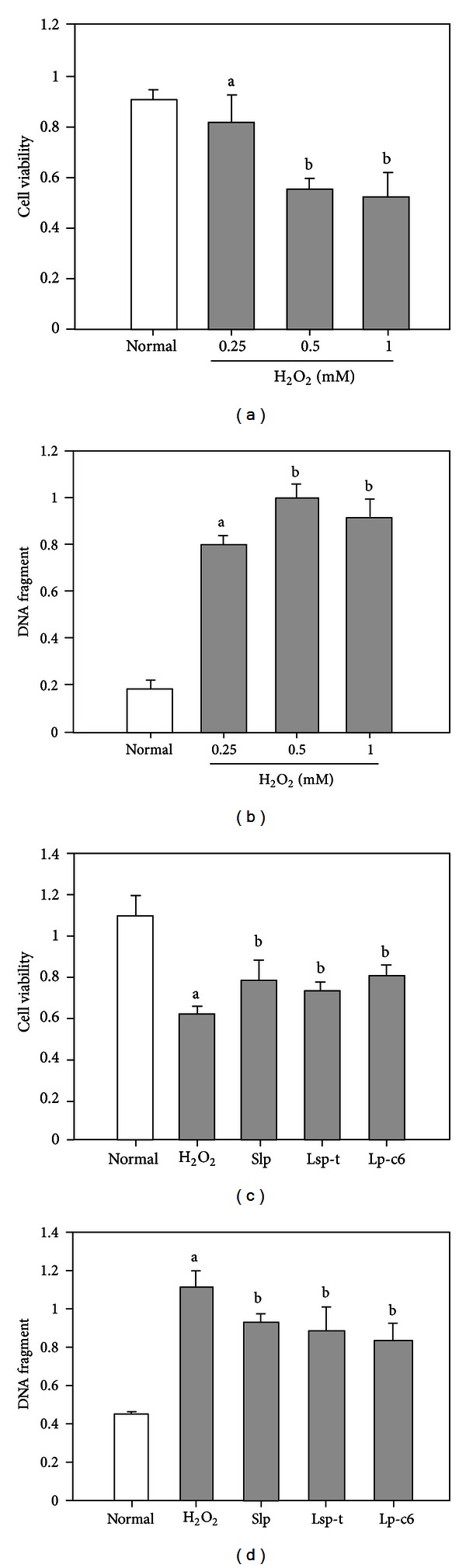
Effects of recombinant Slp and Lsp-t on cell viability and apoptosis in H_2_O_2_-induced HUVEC cells. (a) Cellular viability analysis following treatment of HUVEC cells with different concentrations of H_2_O_2_ as determined by MTT assay. (b) DNA fragmentation analysis in HUVEC cells treated with different concentrations of H_2_O_2_ as determined by ELISA assay. (c) The effects of Slp and Lsp-t on cellular viability in H_2_O_2_-treated HUVEC cells as determined by MTT assay. (d) The effects of Slp and Lsp-t on DNA fragmentation in H_2_O_2_-treated HUVEC cells as determined by an ELISA assay. Purified silkworm lipoprotein 30Kc6 (Lp-c6) was used as a positive control. Different letters refer to the levels of statistical significance at *P* < 0.05. Where the letters match, this refers to no statistical difference between the sets of data.

The effects of recombinant Slp and Lsp-t on cellular viability and apoptosis were further evaluated in H_2_O_2_-treated HUVEC cells. In the first instance, H_2_O_2_ was shown to induce cellular apoptosis in the HUVEC cell model. The dose-dependent effects of H_2_O_2_ on HUVEC cellular activity and apoptosis indicated that H_2_O_2_ decreased cell viability and increased cellular apoptosis (Figures 6(a) and 6(b)). There was a statistically significant difference in respect to cell viability and cell apoptosis between HUVEC cells treated with 0.5 mM H_2_O_2_ and controls. However, there was no further significant decrease in cell viability or increase in cellular apoptosis following treatment with H_2_O_2_ at doses >0.5 mM. Therefore, from the time-response experiments (data not shown) and the data described above, the viability and apoptosis of HUVEC cells were evaluated 24 h after treatment with 0.5 mM H_2_O_2_ for 30 min. 

The effects of Slp and Lsp-t on cell viability and apoptosis in H_2_O_2_-treated HUVEC cells were explored in the following studies. We found that both recombinant Slp and Lsp-t significantly increased cellular viability of H_2_O_2_-treated HUVEC cells as determined by MTT analysis (Figure 6(c)). Both recombinant Slp and Lsp-t significantly decreased DNA fragmentation of HUVEC cells that had been pretreated with H_2_O_2_ (Figure 6(d)). Collectively, these data indicated that both recombinant Slp and Lsp-t have similar biological activities to 30kc6 protein. They could enhance cell viability and inhibit DNA fragmentation in H_2_O_2_-treated HUVEC cells. 

## 4. Discussion

It has been previously reported that several kinds of 30K-family proteins are found in the silkworm, and they mainly serve important roles in energy metabolism and may simultaneously inhibit cellular apoptosis [6–9]. To date, only 3 kinds of 30kD proteins encoded by *30Kc6*, *30Kc12p*, and *30Kc19G1* have been shown to provide energy and serve as antiapoptotic proteins in the silkworm [6–9]. Therefore, we set out to identify new members of the 30K family of proteins. 

 Comparative homology analyses by a bioinformatics approach, demonstrated that both Slp and Lsp-t shared a high degree of amino acid sequence homology with the three reported 30K-family proteins and exhibited a conserved lipoprotein-11 domain. Further analysis revealed that both Slp and Lsp-t had predicted tertiary structures that were similar to those of the reported 30K-family proteins. Therefore, it was reasonable to hypothesize that both Slp and Lsp-t may belong to the 30K-family of proteins. This hypothesis further raised the possibility that both Slp and Lsp-t may demonstrate an antiapoptotic activity. To study the biological function of these two proteins, we used RT-PCR to obtain the cDNA sequences of *Slp* and *Lsp-t* from the fat bodies of the silkworm with the specific aim of expressing and purifying both the Slp and Lsp-t proteins. The results of bioinformatics analysis showed that the cDNAs contained the signal peptides, indicating that they might be expressed as secrete proteins. Whether the signal peptides of Slp and Lsp-t proteins were cleaved or not after expression in BmN cells, further investigations will be needed.

 Previous studies have demonstrated that several 30K-family proteins in the silkworm were highly effective at inhibiting cellular apoptosis in actinomycin-induced Sf9 cells and chemical-treated HeLa cells [6, 8]. Our previous work also indicated that 30K proteins from insects can inhibit apoptosis in H_2_O_2_-stimulated HUVECs [7, 16]. Therefore, whether Slp and Lsp-t exert similar antiapoptotic activities was tested in the present study. H_2_O_2_-induced apoptosis models of BmN and HUVEC cells were first designed and subsequently used to explore the effects of Slp and Lsp-t on cellular viability and apoptosis. Our results demonstrated that both Slp and Lsp-t at a final concentration of 1.5 *μ*g/mL (in BmN cells) and at 4.0 *μ*g/mL (in HUVECs) significantly enhanced cellular viability and decreased DNA fragmentation in both BmN and HUVEC cells that had been pretreated with H_2_O_2_. Our preliminary experiments, results showed that higher concentration of Slp and Lsp-t showed no further protection effects on BmN and HUVEC cellular apoptosis. These data indicated that both Slp and Lsp-t possess important antiapoptotic activities. 

Although several previous studies have demonstrated that silkworm 30K proteins were highly effective at inhibiting cellular apoptosis in insect and mammalian cells [6, 8] the potential antiapoptotic mechanism is still not clear. Kim et al. found that overexpression of 30Kc6 could reduce Caspase 3 activity in vitro study, but in vivo study results showed that 30Kc6 protein did not inhibit Caspase 3 activity indicating that the apoptosis inhibitory activity site of 30Kc6 might exist in the upstream of Caspase 3 [15]. Choi et al. found that 30K protein could inhibit the release of cytochrome c from the mitochondria into the cytoplasm in CHO cells, thus preventing the activation of Caspase 3 as well as its downstream substrates, thereby inhibiting the apoptosis of CHO cell [17].

In summary, both Slp and Lsp-t represent new members of the 30K family of proteins both in terms of their structure and potentially important functions on cellular viability and cell death. Further investigation is needed to investigate the potential antiapoptotic mechanism of Slp and Lsp-t.

## Supplementary Material

The same color indicates the same amino acid residues position of Slp and Lsp-t, respectively.
QMEAN Zscores indicate the prediction of protein's stability.
