# Supplementary material for: Two Novel 30K Proteins Overexpressed in Baculovirus System and Their Antiapoptotic Effect in Insect and Mammalian Cells
Source: Int J Genomics. 2013 Jun 3;2013:323592. doi: 10.1155/2013/323592 (PMC3686079; doi:10.1155/2013/323592)
Supplement: Supplementary file 1 — The same color indicates the same amino acid residues position of Slp and Lsp-t, respectively. QMEAN Zscores indicate the prediction of protein's stability. [file 323592.f1.pdf]

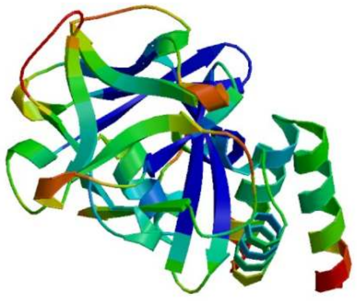

Slp

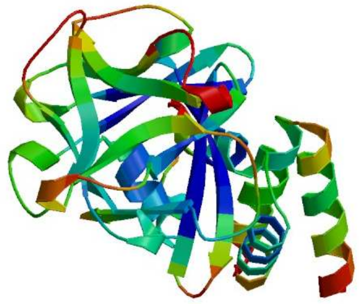

Lsp-t

Slp:

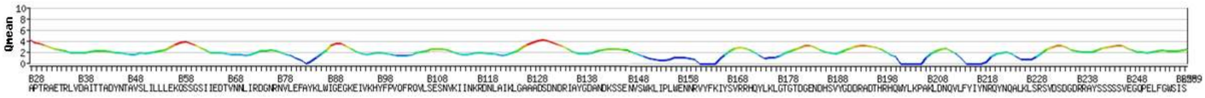

Lsp-t:

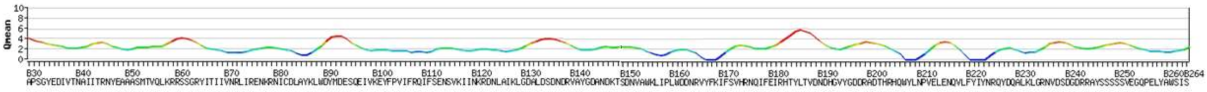

The same color indicates the same amino acid residues position of Slp and Lsp-t, respectively.

QMEAN Zscores indicate the prediction of protein’s stability.
